# Development of extraction and detection method for fluridone in water and sediment by HPLC-UV

**DOI:** 10.1186/s13568-019-0807-4

**Published:** 2019-06-21

**Authors:** Patrick Wickham, Latika Singh, Pramod Pandey, Sarah Lesmeister, Patricia Gilbert, Michael Kwong, Jeffrey Caudill, Jon O’Brien, Sagor Biswas, Swee Teh

**Affiliations:** 1 Department of Population Health and Reproduction, School of Veterinary Medicine, University of California-Davis, 1089 Veterinary Medicine Drive, Davis, CA 95616 USA; 2 Environmental Water Quality and Estuarine Studies, California Department of Water Resources, Sacramento, CA 95691 USA; 3 California Department of Parks and Recreation, Aquatic Invasive Species Branch, Division of Boating and Waterways, Sacramento, CA 95814 USA; 4 Aquatic Health Program, Department of Anatomy, Physiology, and Cell Biology, School of Veterinary Medicine, University of California, Davis, CA 95616 USA

**Keywords:** Herbicide, Fluridone, Water, Sediment, Extraction, Analysis

## Abstract

Fluridone is widely used as a herbicide for controlling invasive aquatic plants such as *hydrilla* in surface water bodies. When applied on surface waters fluridone can attach to bed sediment, requiring rigorous extraction methods prior to analysis. Currently, very limited information exists in terms of fluridone residue detection in delta sediment. In this study, we researched fluridone detection in both water and sediment. To extract fluridone from sediment, here we have tested two extraction methods: (1) a rotavapor method (RM); and (2) a quick, easy, cheap, effective, rugged and safe (QuEChERS) method (QM). The extraction results of RM were compared with those of QM. To quantify fluridone concentrations in extracts, a high-performance liquid chromatography (HPLC)-UV detector was used. HPLC separation was achieved using an Allure C18 5 µm 150 × 4.6 mm column with a mobile phase composed of acetonitrile and water (60:40, v/v). The UV detector was operated at 237 nm. The method was tested and validated using a series of water and sediment samples taken from Sacramento–San Joaquin Delta in California. The average recovery of fluridone was 73% and 78% using RM and QM respectively. The proposed method can be used for testing fluridone in water and sediment samples.

## Introduction

Fluridone, 1-methyl-3-phenyl-5-[3-trifluoromethyl)-phenyl]-4-(1H)-pyridinone, is a herbicide frequently used to control invasive aquatic plant species such as *hydrilla*, *elodea*, and *eichnoria*. These aquatic weeds can be highly invasive and damage aquatic ecosystems by establishing monocultures, outcompeting native species, and clogging waterways (Langeland 1996; Posey et al. 1993) and in order to control them herbicides are often used. As a bleaching herbicide, fluridone inhibits carotenoid synthesis in targeted plant species, preventing photosynthesis and ultimately causing mortality (Bartels and Watson 1978; Netherland and Jones 2015). Though fluridone is not considered a significant health risk to most terrestrial animal species and is not likely to be a human carcinogen (United States Environmental Protection Agency 2004), at high concentrations (> 1000 ppb) in water and riverine sediment it can kill or cause sublethal effects in many adult fish species such as chinook salmon and delta smelt (Paul et al. 1994; Jin et al. 2018).

In general, fluridone is applied in water surface and the concentrations of fluridone in bed sediment are often not known. At lower concentrations (300–1000 ppb) in sediment, fluridone can negatively affect the survivability of fish roe, juvenile fish, aquatic macroinvertebrates, and mollusks (Paul et al. 1994; Archambault et al. 2015; Yi et al. 2011). Further, concentrations from 10 to 300 ppb may cause sublethal effects in juvenile and environmentally sensitive non-target species and can cause mortality in very sensitive species such as water mites (Yi et al. 2011; Siemering 2004).

While a number of studies focused on fluridone detection in water are available (Netherland and Jones 2015), the existing information pertaining to fluridone detection in sediment is limited potentially due to the complexity involved in the extraction and subsequent analysis of particulate-bound fluridone. The concentrations of fluridone in sediment can be particularly important because many fluridone-sensitive species and life stages such as mollusks and fish roe are present in or on sediment (Jacob et al. 2016; Posey et al. 1993; Hamelink et al. 1986) and it is widely acknowledged that fluridone concentrations in sediment can be much higher than in the above water column due to depositional accumulation and the absence of photolysis in bed sediment (Saunders and Mosier 1983; West et al. 1979; Muir et al. 1980). Additional studies are needed describing the suitable methods capable of fluridone detection in sediment with multiple characteristics (i.e., various level of organic matter, clay content, and particle size), and the impacts of various levels of fluridone on fluridone sensitive fish species.

Fluridone is highly lipophilic and therefore bonds strongly to soil and sediment via sorption to particulate organic matter and other bonding sites in the soil matrix (Vassios 2010; Vassios et al. 2011; McCloskey and Bayer 1987). These characteristics make fluridone successful as a long-term invasive plant controller. However, these traits also pose challenges regarding extraction for the purpose of determining concentrations in sediment. As such, an efficient, economic, and accurate methodology for fluridone extraction from sediment is required to monitor fluridone concentrations and persistence. This study was focused on developing a simple solid–liquid extraction method to determine fluridone concentrations in bed sediment.

Simple liquid–liquid and solid–liquid extraction methods involving the extraction of organic compounds from sediment, soil, and plant material using shaking, vortexing, sonication, or other solvent exposure have been in use for more than 100 years (Pfitzner 1895; Trevillian 1930; Othmer 1935). More recently, studies have shown similar methods to be effective at extracting organic pesticides from sediment (Vagi et al. 2007; Weber et al. 2004). As an example, previous authors (Vagi et al. 2007) extracted 17 organic pesticides from oceanic sediment and obtained recoveries ranging from 73.64 to 100% when using ethyl acetate, the same solvent used in this study.

The QuEChERS (Quick, Easy, Cheap, Effective, Rugged, and Safe) method has been utilized for extracting a wide range of emergent compounds from sediment with recovery rates ranging from 40 to 98%. As fluridone is a highly lipophilic compound (Vassios 2010; Vassios et al. 2011), the ability of the QuEChERS method to extract lipid-bound pesticides from fruit and vegetables can be potentially useful in extracting fluridone bound within organic material present in sediment. While multiple studies describing the extraction of multiple pesticides from fruits are available, very few studies (if at all) exist for describing fluridone extraction from wet and submerged sediment. Studies focused solely on Fluridone detection, recoveries, and analyses on delta sediment are yet to be reported.

Therefore, the goal of this study was to develop and test a simple and robust extraction and analysis method for determining fluridone concentrations in the sediment of aquatic environments. Specific objectives of the study were: (1) develop a rotavapor method (RM) for fluridone extraction from sediment with analysis using a high-performance liquid chromatography (HPLC) coupled with UV detector (HPLC-UV) method for calculating fluridone in extracts; (2) test the performance of the QuEChERS method (QM) for extracting fluridone from sediment and compare the results with those of RM; and (3) perform analysis in field samples to determine and compare recoveries of fluridone from RM and QM methods.

## Materials and methods

### Materials and reagents

All HPLC analysis of fluridone was conducted using a Thermo Fisher Dionex UltiMate 3000 Pump, Autosampler, and Diode Array Detector. The HPLC was equipped with a Restek Allure C18 5 μm 150 × 4.6 mm column. To evaporate samples, a Buchi Brinkman Rotovapor and a Thermo-Fisher Reacti-Vap 3 were used. All centrifuging took place in a Fischer Scientific accuSpin 24C clinical centrifuge. HPLC grade water was obtained from Fisher Chemicals (Fisher Scientific, Waltham, MA). Ethyl acetate and acetonitrile solvents were obtained from Sigma-Aldrich (St. Louis, MO). Solid Fluridone (99.8% purity) was also purchased from Sigma-Aldrich for preparing stock solutions and calibration standards. Syringes and 0.22 µm Millex^®^ filters for syringe-driven filtration were purchased from Becton–Dickinson (Franklin Lakes, NJ) and Milipore Sigma (Jaffrey, NH), respectively. QuEChERS EN Method extract pouches and dispersive SPE were obtained from Agilent Technologies (Santa Clara, CA).

### Preparation of standards

Fluridone stock solution (100 ppm) was prepared using HPLC grade water and fluridone solid. This stock solution was used to prepare calibration standards, which were used for method development, calibration, and verification. Using the stock solution, a series of standards with various levels of fluridone concentrations (0.25 ppm, 0.5 ppm, 1 ppm, 1.25 ppm, 2.5 ppm, 5 ppm, 10 ppm, and 25 ppm) were prepared.

### Laboratory sample preparation

Laboratory samples were prepared using sediment from French Island, Sonoma County. The sediment was dried at 30 °C, homogenized, and spiked with Fluridone. To spike fluridone in sediment, 5 g of dried sediment was placed in a 50 mL falcon tube, and 6 mL of fluridone standard was added. A total of 20 samples were spiked for both RM and QM. The first 6 samples were spiked with fluridone levels of 0 ppm (blank), 50 ppb, 250 ppb, 500 ppb, 1000 ppb, and 5000 ppb to determine method linearity. An additional 7 samples were spiked with 10,250 ppb to test the recovery at high concentrations. A final set of 7 samples spike with 500 ppb were used to determine recovery at lower concentrations as well as limit of detection (LOD) and limit of quantification (LOQ). Limit of detection (LOD) and limit of quantification (LOQ) were both determined using a conservative numerical method (1) (Shrivastava and Gupta 2011).

1 $$LOD = \frac{{3.3\upsigma}}{\text{S}}\quad and\quad LOQ = \frac{{10\upsigma}}{\text{S}}$$

where S = the slope of the regression between the spiked lab concentration (ppm) and corresponding area (CC), and $\upsigma$ = the standard deviation.

### Field sample collection and preparation

Field samples were obtained from multiple locations (seven sites) in the California delta. Sampling sites were located within Sonoma County, in a tidally-influenced freshwater riverine environment. At each location, a sediment sample was collected via a sediment dredge sampler. Each sediment sample weight was around 2 kg.

### Rotavapor extraction methodology

A wet sediment mass of 10 g was placed in a 50 mL centrifuge tube. 1 mL of ethyl acetate/g of sediment was added. The sample was then vortexed for 5 min, sonicated for 5 min, and vortexed for a further 5 min. Vortexing allows for all the solvent to interact with and extract organic compounds from the water and sediment matrix. Sonication serves to break down any soil aggregates that may persist after the initial vortexing. Samples were then centrifuged at 4500 rpm for 5 min to separate the soil particulates, water, and organic solvent. All the ethyl acetate supernate was then separated into a separate vial, while the remaining water and sediment was discarded. This supernate was then evaporated down to 1 mL using a Buchi Brinkman Rotavapor with a bath temperature of ≈ 35 °C. The rotavapor was equipped with an Edwards vacuum pump with a maximum vacuum of − 2 mmHG. Each round bulb utilized for evaporation was washed with ≈ 3 mL of ethyl acetate to ensure all fluridone was transferred to the next evaporation stage and to avoid carry-over between samples. At this stage, samples consisted of ≈ 4 mL of ethyl acetate. These samples were filtered using a syringe-driven 0.22 µm Millex SLGV013SL filter. Samples were then transferred into 4 mL vials and evaporated entirely using a Thermo-Fisher Reacti-Vap 3. Samples were then reconstituted in 2 mL acetonitrile and vortexed for 5 min. A 200 µL aliquot of the sample was then placed in a 2 mL vial with a riser for HPLC-UV analysis. As the rotavapor technique concentrates the solution down to 2 mL, the resultant concentration was high and required correction using a dilution factor. This dilution factor was obtained by simply dividing the initial volume of spiked water added to the sediment sample by the final volume of ACN prepared for HPLC analysis (Table 1).

**Table 1:** Descriptions of sample and solvent volume and dilution/concentration used for rotavapor (RM) and QuEChERS (QM) based analysis

Rotavapor	H_2_O (mL)	Sediment (g)	Acetonitrile (mL)	Final solvent vol. (mL)	Dilution/concentration factor
Lab	6	5	11	2	3
Field	–	40	20	2	20

QuEChERS	H_2_O (mL)	Sediment (g)	Acetonitrile (mL)	Final solvent vol. (mL)	Dilution/concentration factor
Lab	6	5	11	11	0.545
Field	–	10	10	10	1

### QuEChERS extraction methodology

The wet sediment sample of 10 g was then placed in a 50 mL falcon (centrifuge) tube. To this 10 mL of acetonitrile was added, and vortexed for 5 min. To each sample an EN Method QuEChERS Extract Pouch was added, containing 1 g sodium chloride, 4 g magnesium sulfate, 1 g sodium citrate, and 0.5 g sodium hydrogencitrate sesquihydrate. Samples were immediately shaken and vortexed for 2 min after which they were centrifuged for 5 min at 4500 rpm to separate the organic solvent from the water and sediment matrix. A 1 mL aliquot of the supernate was then removed and placed in an EN Method QuEChERS Dispersive SPE 2 mL Fatty Samples vial containing 25 mg PSA, 25 mg C18EC, and 150 mg magnesium sulfate. The vial was then vortexed for 1 min and centrifuged for 3 min at 4500 rpm. 1 mL of the supernate was filtered using a syringe-driven 0.22 µm filter and placed in 2 mL vials for HPLC analysis. As QM dilutes the solution to 11 mL, the resultant concentration was low and required correction using a concentration factor. This concentration factor was obtained by simply dividing the initial volume of spiked water added to the sediment sample by the final volume of ACN prepared for HPLC analysis (Table 1).

### Operating conditions of HPLC-UV detectors

**Fig. 1:**
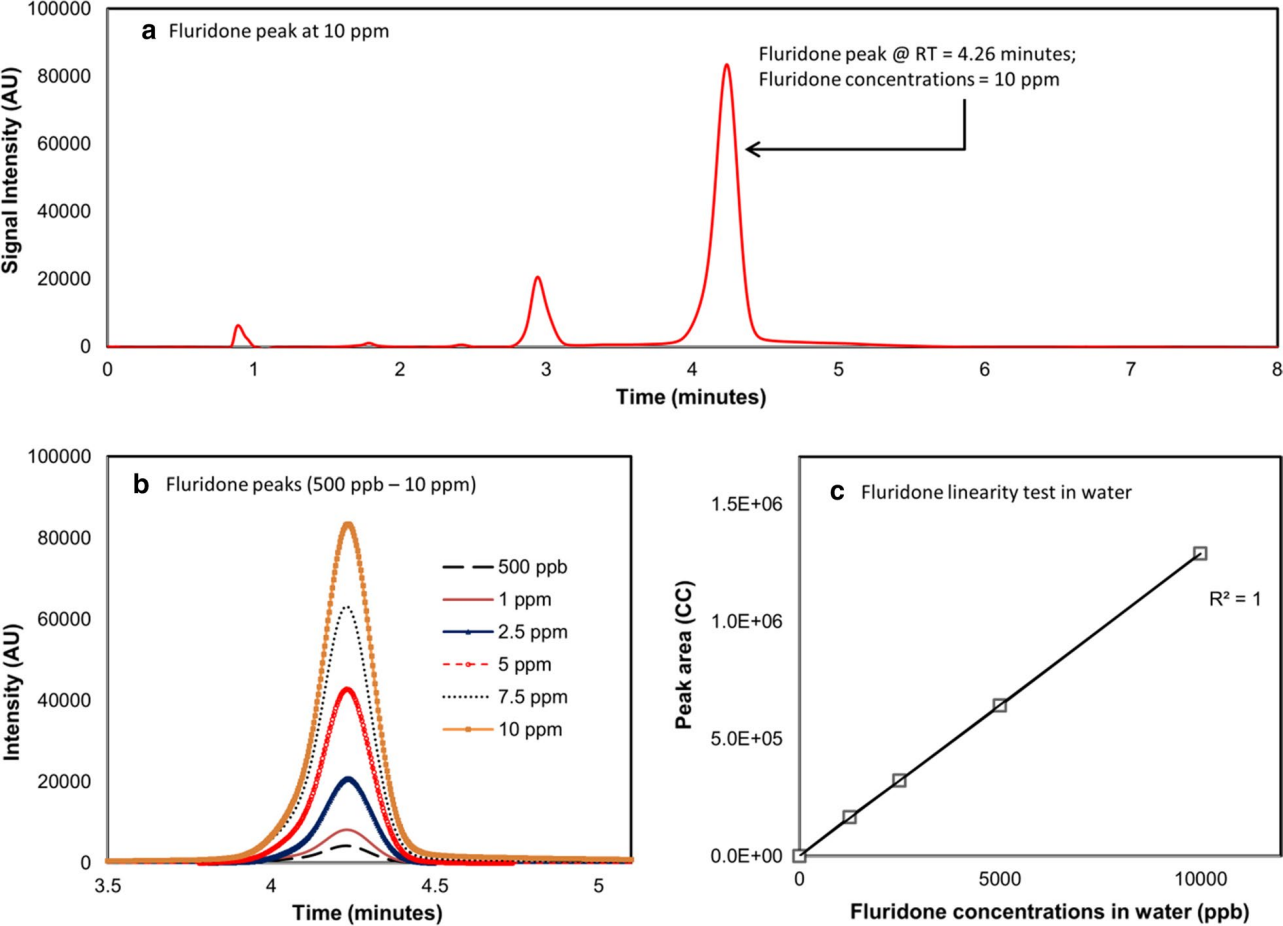
Fluridone detection in water samples: **a** fluridone peak at 10 ppm; **b** fluridone peaks at 500 ppb–10 ppm; and **c** Relationship between fluridone concentrations in water and peak area

All HPLC analysis was carried out on a Fisher UltiMate 3000 equipped with a Restek Allure C18 5 μm 150 × 4.6 mm column. Acquisition wavelength was set at 267 nm and the carrier solvent was acetonitrile and water (60:40) with a flow rate of 1 mL/min. Sample injection volume was 30 µL. Oven temperature was 26.0 °C and the total sample retention time was 8 min. This methodology resulted in consistent fluridone peaks at 4.21–4.26 min of sample retention (Fig. 1a). All samples were analyzed using Xcalibur chromatography software.

## Results

### Fluridone HPLC detection and linearity

All standards created for calibration curves exhibited excellent chromatography in ACN and water. In both solvents, the retention time of fluridone was constant at 4.26 min. Concentrations from 0.5 to 10 ppm did not shift this retention time (Fig. 1b). Due to the use of ACN as the primary carrier solvent, the retention time seen here is faster than what has been observed in similar methods that utilize methanol as the primary carrier solvent (Netherland et al. 2002; Fox et al. 1991; Getsinger et al. 2002). This allows for a shorter total HPLC run time, minimizing solvent use and expediting analysis. All calibration curves prepared exhibited *R*^*2*^ = 0.99 in both water and ACN (Fig. 1c), with the majority displaying R^*2*^ = 1. These results are consistent with the linearity seen in previous studies using similar methodologies (Netherland et al. 2002; Fox et al. 1991).

### Fluridone analysis in sediment using RM

**Fig. 2:**
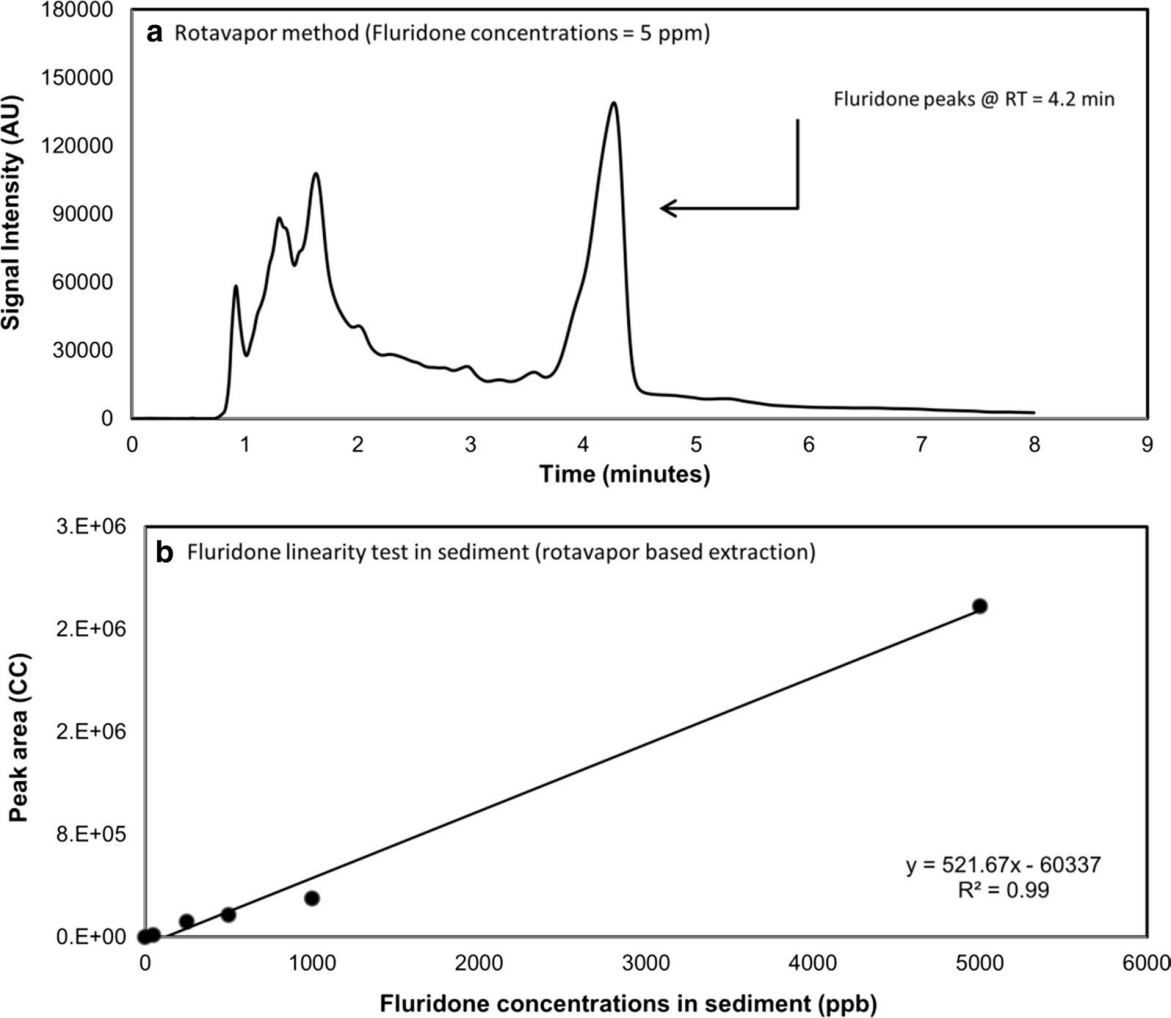
Fluridone detection in sediment samples using rotavapor method (RM): **a** fluridone peak in sediment sample at 5 ppm; and **b** relationship between fluridone concentrations in sediment and peak area

Laboratory samples extracted using RM resulted in consistent fluridone retention times around 4.2 min (Fig. 2a). The RM achieved an average recovery of 59.99% (± 13.46%) when analyzing the samples intended for linearity testing (50–5000 ppb). In the samples ran at low concentration (500 ppb), the RM method achieved an average of 60.20% recovery (± 12.00%). In the seven samples ran at 10,250 ppm, this method achieved an average of 95.41% recovery (± 11.61%). Overall, this method achieved an average of 73.90% recovery (± 20.10%) (Table 2). RM also displayed very good UV response linearity (*R*^*2*^= 0.99) (Fig. 2b).

**Table 2:** Recovery of fluridone, limit of quantification, and limit of detection on rotavapor and QuEChERS based analysis

	Sample name	Spiked fluridone concentrations in samples (ppb)	Detected signal area (CC)	Observed fluridone concentrations by HPLC	Recovery percentage (%)
Rotavapor	R1	500	94,965	203.485	40.7
	R2	500	177,633	396.377	79.3
	R3	500	146,492	323.715	64.7
	R4	500	141,065	311.052	62.2
	R5	500	116,953	254.790	51.0
	R6	500	141,997	313.226	62.6
	R7	500	138,216	304.404	60.9
QuEChERS	Q1	500	31,208	301.204	60.2
	Q2	500	34,901	348.637	69.7
	Q3	500	32,750	321.009	64.2
	Q4	500	31,746	308.114	61.6
	Q5	500	28,687	268.824	53.8
	Q6	500	30,911	297.389	59.5
	Q7	500	32,553	318.479	63.7

	Slope (S)	Std. deviation (σ)	LOD (3.3σ/S)	LOQ (10σ/S)	
Rotavapor	521.66	25,666.23	162.36	492.00	
QuEChERS	77.86	1911.22	81.00	245.45	

### Fluridone analysis in sediment using QM

**Fig. 3:**
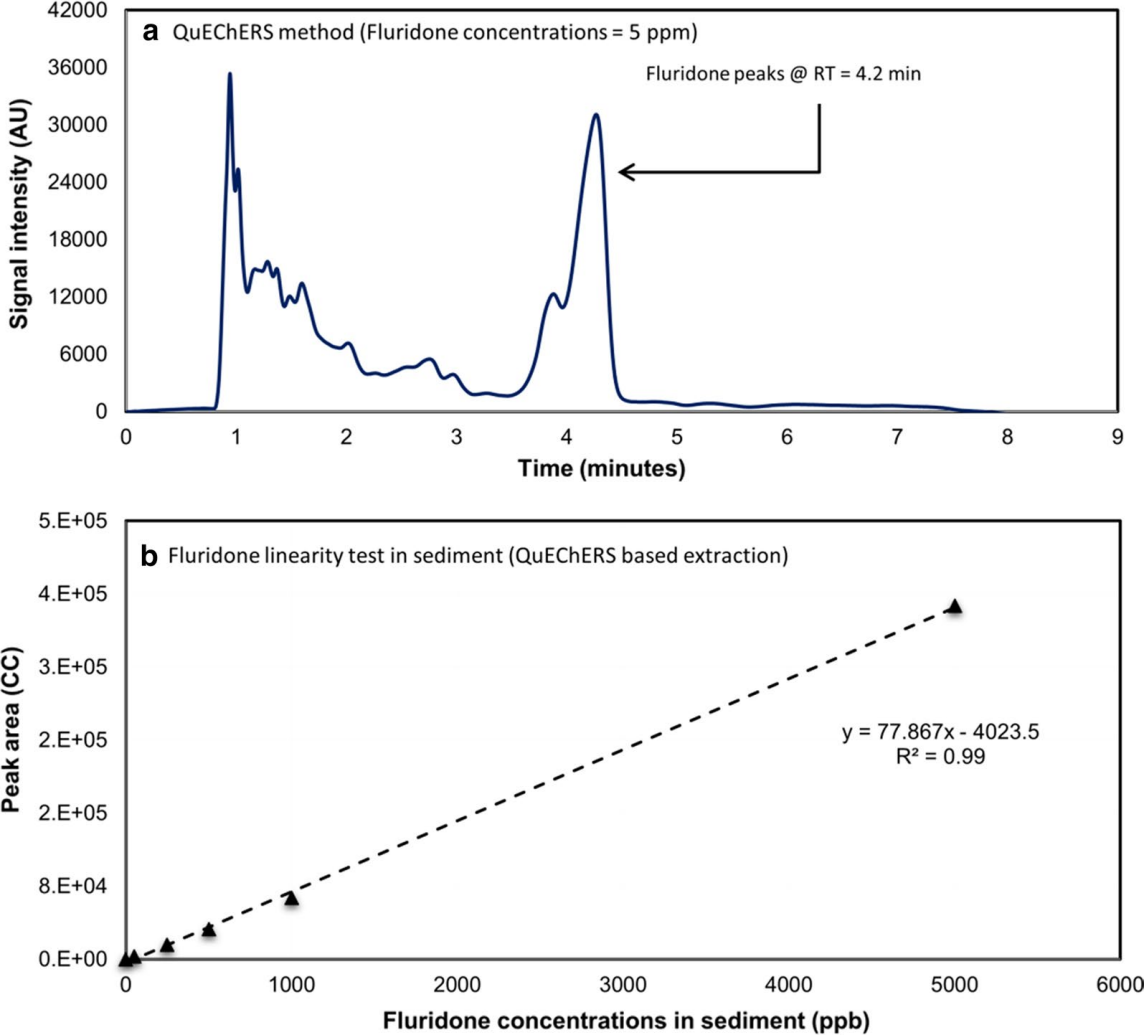
Results of fluridone detection using QuEChERS (QM): **a** fluridone peak in sediment sample at 5 ppm; and **b** relationship between fluridone concentrations in sediment and peak area

Laboratory samples extracted using QM resulted in consistent fluridone retention times around 4.2 min (Fig. 3a). Samples processed using QM achieved an average recovery of 66.48% (± 16.24%) when analyzing the samples intended for linearity testing (50–5000 ppb). In the seven samples ran at 500 ppb, this method achieved an average of 61.80% recovery (± 4.90%). In the seven samples ran at 10,250 ppm, this method achieved an average of 104.04% recovery (± 7.21%). Overall, the method achieved an average of 78.60% recovery (± 22.05%) (Table 2). QM displayed excellent UV response linearity (*R*^*2*^= 0.99) (Fig. 3b).

### Fluridone measurements in field samples

Both methodologies were able to extract fluridone from the field samples and were well correlated with each other (*R*^*2*^ = 0.835, p < 0.001) (Fig. 5a, b). The average and median concentration after Rotavapor analysis were 112.6 ppb and 45.9 ppb, respectively, while the average and median QuEChERS concentrations were 112.78 ppb and 68.3 ppb, respectively. These data slightly favor the QuEChERS recovery but the trend is not statistically significant, which supports the conclusions found in lab samples.

### LOD, LOQ, sensitivity, and method robustness

**Fig. 4:**
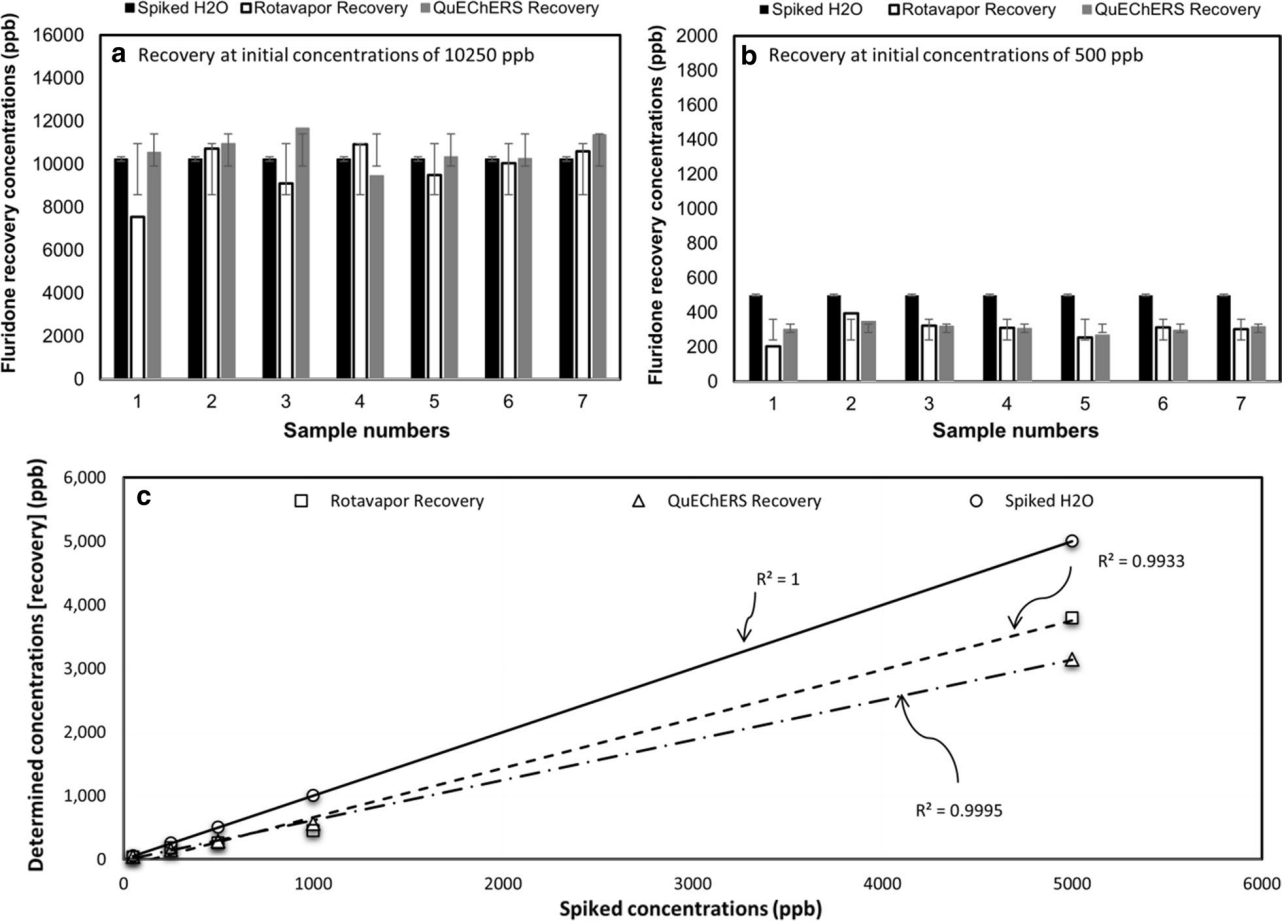
Results of spiked water, rotavapor method (RM) recovery, and QuEChERS Method (QM) recovery: **a** recovery at high concentrations (10,250 ppb); **b** recovery at low concentrations (500 ppb); and **c** linearity test at various concentrations of fluridone in water and sediment

**Fig. 5:**
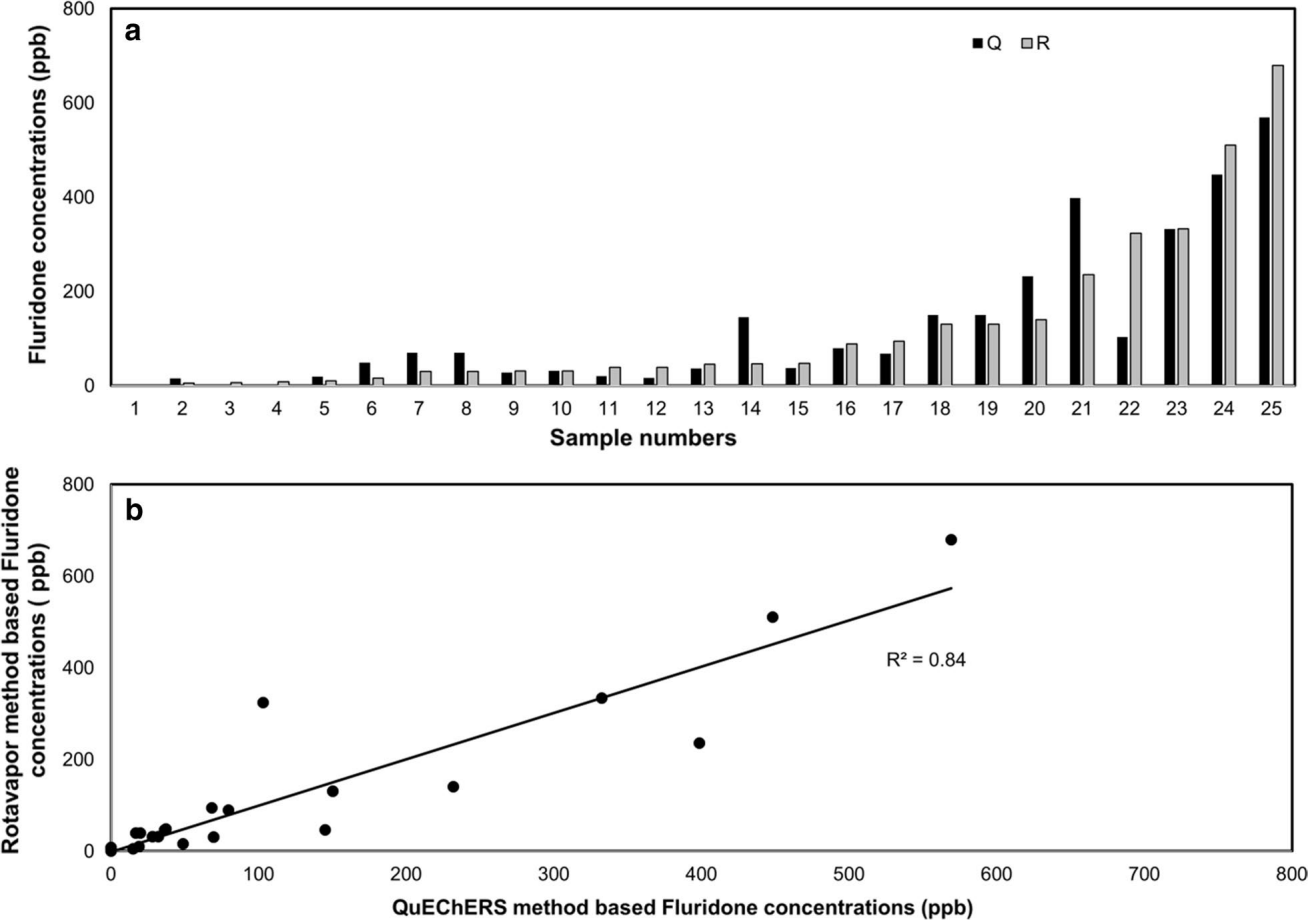
Relative performance of sediment extractions methods on field samples: **a** fluridone concentrations in sediment [*Q* QuEChERS method, *R* rotavapor method]; **b** comparison between results of rotavapor method (RM) and QuEChERS method (QM)

The RM has approximately double the LOD and LOQ compared with the QM, likely due to the absence of any SPE cleanup step to reduce background noise generated from the complex soil matrix, which increased standard error. However, the RM, LOD and LOQ are still within acceptable ranges for determining fluridone concentrations in sediment, as concentrations below 162 ppb are less likely to cause major effects in most species (Yi et al. 2011; Siemering 2004). Additionally, chromatographic peaks corresponding to concentrations below these limits were quantified in both field and lab samples and exhibited good linearity (Figs. 4c; 5a).

To test the robustness and replicability of the HPLC method, three water samples spiked at around 5 ppb were analyzed using the HPLC methodology but with adjustments to flow, column temperature, and mobile phase constitution. Specifically, flow was ran at 0.8 mL/min, 1 mL/min, and 1.2 mL/min; column temperature was ran at 26 °C, 28 °C, and 30 °C; and the mobile phase was ran at 50:50 ACN/H_2_O, 70:30 ACN/H_2_O, and 80:20 ACN/H_2_O.

Flow rate does appear to significantly affect both peak occurrence and peak area (p < 0.001), with faster flow causing peaks to occur earlier and with a smaller area. Column temperature does not appear to affect peak area (p = 0.547) but does affect peak occurrence (p < 0.001), with higher temperatures causing earlier peak occurrence. Mobile phase constitution greatly influenced peak occurrence (p < 0.001) and to much a lesser extent affected peak area (p = 0.17), with a higher proportion of ACN causing peaks to arrive earlier and with slightly larger area (Table 3).

**Table 3:** Sensitivity and robustness of fluridone detection method

Parameters	Change in parameter values	(min)					Peak area counts				
		Run 1	Run 2	Run 3	Avg.	Std. dev.	Run 1	Run 2	Run 3	Avg.	Std. dev.
Flow (mL/min)	1	4.28	4.33	4.33	4.31	0.03	92,311	92,398.00	92,005.00	92,238.00	206.42
	0.8	5.41	5.41	5.41	5.41	0.00	115,100.00	115,346.00	114,780.00	115,075.33	283.81
	1.2	3.65	3.64	3.64	3.64	0.01	76,440.00	77,136.00	76,539.00	76,705.00	376.52
Temperature (°C)	28	4.29	4.29	4.29	4.29	0.00	92,979.00	93,011.00	93,520.00	93,170.00	303.53
	26	4.32	4.33	4.33	4.33	0.01	93,687.00	90,813.00	91,629.00	92,043.00	1481.05
	30	4.26	4.25	4.26	4.26	0.01	92,054.00	92,344.00	93,267.00	92,555.00	633.43
Mobile phase (Acetonitrile:H_2_O) (%)	50:50	8.25	8.27	8.27	8.26	0.01	93,517.00	92,177.00	93,384.00	93,026.00	738.26
	70:30	2.94	2.83	2.83	2.87	0.06	90,582.00	91,803.00	93,268.00	91,884.33	1344.85
	80:20	2.17	2.11	2.11	2.13	0.03	90,124.00	91,063.00	90,963.00	90,716.67	515.69

## Discussion

The RM and QM produced similar results in both field and lab scale studies (Fig. 4a–c). While QM exhibited about 5% higher recovery on average, the differences in recovery between RM and QM were not statistically significant (p ≈ 0.75), suggesting that the RM is comparable to the QM. Additionally, both methods exhibited recoveries comparable with results from other current pesticide extraction techniques, and certain differences in recoveries are expected because of the differences in extraction methods. While RM takes a relatively longer time, it is less expensive than QM. Recent studies have exhibited recoveries from sediment and soil ranging from less than ~ 40 to over 100% (Nannou et al. 2018; Miyawaki et al. 2018; Zhao et al. 2018). Previous results showed that depending on the compound and extraction process involved, the recoveries percentages change considerably (Vagi et al. 2007; Weber et al. 2004). In a previous study, 100 soil samples obtained from multiple southern districts of Jordan during 2016 and 2017 to monitor pesticides residue using LC–MS/MS (Kailani et al. 2019). The method was used to analyze 448 pesticides, and the recoveries were between 70 and 120%. To authors’ knowledge, very limited information focused on quantifying fluridone residue analyses in sediment exist in public domain, which emphasizes the importance of this study. Although, multiple studies describing pesticide extraction methods for fruits are available. The recoveries of pesticides by LC–MS/MS varied between 73.45 and 112%, while pesticide recoveries from GC–MS/MS method varied 79.62–116.65%.

Results of this study, and previous studies (Paul et al. 1994; Archambault et al. 2015; Yi et al. 2011) showed that sediment can be a considerable source of herbicides, which can re-suspend in water column during increased flow and tide conditions. Results of both methods (RM and QM) showed the elevated level of herbicides in sediment. In terms of the extraction efficiencies, the RM and QM performance was consistent in both sediment and water samples of lab studies and field studies. The RM method produced results comparable to the QM, with only 5% lower average recovery. The extraction recovery with RM averaged 73.09% (± 20.97%), while the recovery with QM averaged 78.06% ± 22.05%. The LOD and LOQ of the RM method were 162.36 and 492.00 ppb respectively, which was approximately half the LOD and LOQ of the QM method (81.00 ppb and 245.45 ppb, respectively), but still within the desired range. When processing field samples, both the RM and QM methods were positively correlated with each other (*R*^*2*^ = 0.835), generating average concentrations of 122.61 ppb (± 171.38 ppb) and 122.68 ppb (± 155.02 ppb), respectively. While QM is considerably faster than RM, the RM can be more cost effective as it requires relatively less expensive supplies.

In this study, results of both the RM and QM methods were improved when analyzing the spiked samples (lab samples) containing very high concentrations of fluridone (Fig. 4a, b). In high concentrations, it is likely that many of the potential bonding sites within the sediment matrix are already filled (i.e., saturated) by fluridone, and therefore much of the fluridone remains unbonded to particulate bonding sites (Langeland 1996; Miyawaki et al. 2018). As it is generally easier for solvents to capture organic compounds in aqueous solution compared with particulates, it is expected that these concentrations had better recovery in lab samples with high concentrations of fluridone (Nannou et al. 2018; Weber et al. 2004).

In addition, any chromatographic noise produced by the complex sediment composition is minimized by the large peaks observed in samples with high concentration. In the seven samples ran at 10,250 ppb, many samples obtained recoveries greater than 100%, after both extraction methods (Fig. 4a). For example, if 11 mL of acetonitrile evaporates to 10.5 during the extraction process, a recovery of 100% would appear as 104.7% after analysis. Some errors may occur while calculating HPLC peak areas, which are inherent limitations of chromatographic peak area calculation. In the sediment prepared for laboratory samples there was no background fluridone, therefore all laboratory sediment blanks had no chromatographic peak corresponding with fluridone. While both RM and QM established excellent linearity at multiple concentrations, QM method yielded slightly higher correlation coefficient values (*R*^*2*^ = 0.9993) than RM (*R*^*2*^ = 0.9928) (Fig. 4c). While QM produced about 5% higher average recovery and exhibited slightly better linearity, the differences were not statistically significant (p > 0.5). This suggests both RM and QM methods can be used for the extraction of fluridone from sediment.

The recoveries of fluridone are likely to change depending on the organic matter content, sediment characteristic, and environment from where sediment was obtained. Sorption process controls the extraction and mobility of herbicides in soil and sediment (Morillo et al. 2004; Sun et al. 2012) and the content of organic matter in sediment plays a crucial role in herbicide sorption (Chefetz and Xing 2009; Cornelissen et al. 2005). Further attachment and release of pesticides from soil and sediment depends of multiple physicochemical characteristics such as surface area and volume (Sabljic et al. 1995; Mamy and Barriuso 2005). Due to the fact that sediment characteristics (i.e., organic matter, particle size, clay content, and moisture) changes substantially with the source of sediment, and it plays an important role in attachment and detachment of fluridone in sediment, certain deviation in results among various studies reporting herbicide recovery are expected.

Compared to lab samples, field samples displayed greater variability in recovery among methods. The increased variability in field samples is likely due to the large sample size (of which an aliquot was taken for processing by each method) and the influence of environmental factors such as sediment heterogeneity and the presence of relatively larger particles of organic matter such as leaves residues (Nannou et al. 2018; Saunders and Moser 1983; Shrivastava and Gupta 2011; McCloskey and Bayer 1987). In lab samples, the process was controlled, creating a low possibility for error. Despite this, both methods were successfully utilized to extract fluridone from wet field sediment and were consistent with one another.

A previous study reported multiresidue method for determination of pesticides in fruits and vegetables (QuEChERS method) (Schenck et al. 2008). Authors compared single solid-phase extraction (SPE) column cleanup and dispersive cleanup used in QuEChERS method. Recovery data of samples with pesticides level varying from 20 ppb to 1.0 ppm showed that SPE cleanup method was better than dispersive method. The modification on QuEChERS method was suggested to improve the extraction.

While evaluating the fluridone residue levels in sediment, the concentrations present in some of the field samples are well within the range of concentrations which may prove harmful to aquatic life (Yi et al. 2011; Siemering 2004). At least two of the samples exhibited concentrations above 500 ppb without accounting for recovery, meaning that actual concentrations may be as high as 600–800 ppb. The presence of field samples containing these high amounts of fluridone further validate previous studies which suggest fluridone may accumulate in the sediment of ambient water bodies after successive applications (Saunders and Mosier 1983; West et al. 1979; Muir et al. 1980). In general, sampling and testing of water column is used to determine the presence of herbicide residues in water bodies, and involving the impacts of sediment bound herbicides on water column can further assist in improving the understanding of herbicide loads and residues present in a waterbody.

## Data Availability

All data supporting the results & discussion, and conclusions of this study are included in the manuscript.
